# Performance Evaluation of VIDAS^®^ Diagnostic Assays Detecting Anti-Chikungunya Virus IgM and IgG Antibodies: An International Study

**DOI:** 10.3390/diagnostics13132306

**Published:** 2023-07-07

**Authors:** Geovana M. Pereira, Erika R. Manuli, Laurie Coulon, Marina F. Côrtes, Mariana S. Ramundo, Loïc Dromenq, Audrey Larue-Triolet, Frédérique Raymond, Carole Tourneur, Carolina dos Santos Lázari, Patricia Brasil, Ana Maria Bispo de Filippis, Glaucia Paranhos-Baccalà, Alice Banz, Ester C. Sabino

**Affiliations:** 1 Instituto de Medicina Tropical, Faculdade de Medicina da Universidade de São Paulo, São Paulo 05403-000, Brazil; geovanapereira16@gmail.com (G.M.P.); erikamanuli@gmail.com (E.R.M.); marinafarrel23@gmail.com (M.F.C.); marianasevero@gmail.com (M.S.R.); glaucia.baccala@biomerieux.com (G.P.-B.); 2 Faculdade de Medicina da Universidade Municipal de São Caetano do Sul, São Paulo 09521-160, Brazil; 3 Laboratório de Investigação Médica/Parasitologia LIM/46, Hospital das Clínicas da Faculdade de Medicina da Universidade de São Paulo, São Paulo 05403-010, Brazil; 4 bioMérieux, 69280 Marcy l’Etoile, France; lauriecoulon11@gmail.com (L.C.); loic.dromenq@biomerieux.com (L.D.); audrey.larue@me.com (A.L.-T.); frederique.raymond@biomerieux.com (F.R.); carole.tourneur@biomerieux.com (C.T.); alice.banz@biomerieux.com (A.B.); 5 Hospital das Clínicas da Faculdade de Medicina da Universidade de São Paulo, São Paulo 05403-010, Brazil; carolina.lazari@hc.fm.usp.br; 6 Instituto Nacional de Infectologia Evandro Chagas, Fundação Oswaldo Cruz, Fiocruz, Rio de Janeiro 21040-360, Brazil; patricia.brasil33@gmail.com; 7 Laboratório de Arbovírus e Vírus Hemorrágicos, Instituto Oswaldo Cruz/Fiocruz, Rio de Janeiro 21040-360, Brazil; ana.bispo@ioc.fiocruz.br

**Keywords:** chikungunya virus, CHIKV, VIDAS, enzyme-linked immunosorbent assay, ELISA, IgM, IgG, capture immunoassay, enzyme-linked fluorescent assay, ELFA

## Abstract

Chikungunya (CHIK) is a debilitating mosquito-borne disease with an epidemiology and early clinical symptoms similar to those of other arboviruses-triggered diseases such as dengue or Zika. Accurate and rapid diagnosis of CHIK virus (CHIKV) infection is therefore challenging. This international study evaluated the performance of the automated VIDAS^®^ anti-CHIKV IgM and IgG assays compared to that of manual competitor IgM and IgG ELISA for the detection of anti-CHIKV IgM and IgG antibodies in 660 patients with suspected CHIKV infection. Positive and negative agreements of the VIDAS^®^ CHIKV assays with ELISA ranged from 97.5% to 100.0%. The sensitivity of the VIDAS^®^ CHIKV assays evaluated in patients with a proven CHIKV infection confirmed reported kinetics of anti-CHIKV IgM and IgG response, with a positive detection of 88.2–100.0% for IgM ≥ 5 days post symptom onset and of 100.0% for IgG ≥ 11 days post symptom onset. Our study also demonstrated the superiority of ELISA and VIDAS^®^ assays over rapid diagnostic IgM/IgG tests. The analytical performance of VIDAS^®^ anti-CHIKV IgM and IgG assays was excellent, with a high precision (coefficients of variation ≤ 7.4%) and high specificity (cross-reactivity rate ≤ 2.9%). This study demonstrates the suitability of the automated VIDAS^®^ anti-CHIKV IgM and IgG assays to diagnose CHIKV infections and supports its applicability for epidemiological surveillance and differential diagnosis in regions endemic for CHIKV.

## 1. Introduction

Chikungunya (CHIK) is a debilitating disease caused by the chikungunya virus (CHIKV) and transmitted to humans by *Aedes* spp. mosquitoes [1]. CHIKV was first identified in 1952 in Tanzania [2] and has now spread to over 100 countries across Africa, Asia, Europe, and the Americas, with multiple outbreaks affecting millions of people [1,3,4,5,6]. This alarming increase in CHIKV spread is likely of multifactorial origin, including virus and vector adaptation to changes in the environment and human behaviour, and enhanced global dissemination due to increased urbanisation and international travel [1,7]. Phylogenetic studies identified four main CHIKV genotypes, namely the (i) ‘East Central South African’ (ECSA), (ii) ‘West African’ (WA), (iii) ‘Asian’, and (iv) the recently emerged, ECSA-diverged ‘India Ocean’ lineage (IOL) [1,3,5,6]. Although studies directly comparing the virulence of these geographic genotypes are scarce, a few investigations have suggested that CHIKV lineages present differences in their transmission cycles and that some genotypes might be preferentially associated with a higher prevalence of self-reported long-term chronic CHIKV symptoms [1,3,6].

Like dengue virus (DENV) and Zika virus (ZIKV), CHIKV is a single-stranded RNA arbovirus with similar epidemiology and transmission cycles [8]. Accordingly, co-circulation of these arboviruses in overlapping endemic regions and co-infection cases have been described [7,8,9,10,11]. Moreover, following infection by these arboviruses, clinical symptoms at disease onset are often similar and clinically non-specific, including fever, headache, myalgia, arthralgia, and maculopapular rash [7,8,12,13]. This raises the challenge of CHIKV diagnosis and emphasises the need for efficient strategies of epidemiological surveillance and differential diagnosis [7,8,10,11,13,14].

Following CHIKV infection, the incubation period ranges from 1 to 12 days. The early acute phase of infection is usually characterised by a sudden onset of high fever (in 85% of patients), correlating with the presence of elevated CHIK viral load in the blood. The onset of fever is followed by intense polyarthralgia, which can last two to three weeks, and a rash (in 40–75% of patients). In the post-acute phase (>3 weeks to 3 months), clinical manifestations, notably joint pain, persist in more than half of the patients. When symptoms have not subsided after 3 months, the patient enters the chronic phase of the disease (>3 months to several years, affecting 40–80% of patients). The chronic disease can progress to (i) cure without sequelae, upon treatment or spontaneously, (ii) further persistence of joint and/or other symptoms, or (iii) aggravation because of exacerbation of inflammatory and/or degenerative processes [1,5,6,15,16].

In addition to these typical clinical manifestations, atypical features and complications might occur, such as neurologic disorders, notably in infected individuals with comorbidities, and according to age (the elderly and infants) [1,7,12]. Altogether, despite a low mortality rate, the morbidity associated with CHIKV infection is high, and CHIK illness can be severe and durably debilitating [1].

No specific antiviral therapy exists for acute CHIKV infection, and patient management relies mainly on supportive care to treat pain and inflammatory symptoms [1,7,12,16,17]. Despite sharing similar clinical manifestations at illness onset, which is associated with a risk of misdiagnosis, the course of disease following infection by distinct arboviruses such as CHIKV, DENV, and ZIKV varies greatly. Therefore, a reliable and accurate early diagnosis is essential to ensure proper patient management, while adopting timely preventive measures and implementing suitable epidemiologic surveillance [7,8,10,11,13,14].

Current recommendations [13,15,17,18] for the confirmation of CHIKV infection in a suspected case (i.e., with the characteristic triad of fever, rash, and joint manifestations) are based on the kinetics of CHIKV viremia and of the host immune response, with the detection of CHIKV RNA by means of real-time reverse transcription-polymerase chain reaction (rRT-PCR) within the first week (≤7 days) of symptom onset, and detection of anti-CHIKV immunoglobulin M (IgM) and/or IgG thereafter (>7 days). rRT-PCR alone is usually recommended between day 0 and 5 post symptom onset, rRT-PCR and anti-CHIKV IgM serology between day 5 and 7, and serology only after day 7. A positive rRT-PCR is confirmatory of an acute CHIKV infection, while a positive anti-CHIKV IgM test is presumptive of a CHIKV infection. Seroconversion or a 4-fold rise in anti-CHIKV IgG measured in paired serum samples collected in the acute and post-acute (convalescent) phases (two to three weeks apart) also indicates an active CHIKV infection. Given that CHIKV-specific IgG can be detected several years after the initial infection, seroconversion/antibody rise also allows us to rule out a past infection. In the chronic phase, as for the post-acute phase, CHIKV serology should confirm the diagnosis together with a biological evaluation of inflammatory rheumatism. Finally, in the case of negative rRT-PCR and serology in acute samples, serology should be repeated on convalescent-phase samples to definitively rule out CHIKV diagnosis [1,5,6,7,13,15,17,18,19,20].

rRT-PCR assays, both as singleplex (CHIKV RNA) or multiplex (e.g., differential screening of CHIKV, DENV, and ZIKV RNAs), have proven to be highly sensitive and specific, although no molecular gold standard exists to date [5,10,14,19]. rRT-PCR tests present, however, the potential caveat that not all existing assays detect all known CHIKV genotypes [6,19], an issue not shared by existing anti-CHIKV antibody detection tests due to the demonstrated cross-reactivity against heterogenous CHIKV genotypes [6,21]. Numerous serological tests detecting CHIKV-specific IgM and IgG antibodies have been developed and commercialised [5,6,10,11,19,20,22,23,24]. Enzyme-linked immunosorbent assays (ELISA) have demonstrated acceptable performance for the detection of anti-CHIKV IgM and IgG. In comparison, rapid diagnostic tests (RDT) showed very low sensitivity and specificity for the detection of CHIKV-specific IgM and IgG antibodies [5,6,10,11,12,19,20,22,23,24]. Despite demonstrating good performance, ELISAs are manual and time-consuming (about 1.5–2 h per test) methods, which might represent a burden for testing laboratories at times of epidemic outbreaks. The implementation of an automated system allowing rapid execution and interpretation of results would be of clear benefit.

VIDAS^®^ anti-CHIKV IgM and IgG assays are fully automated CE-marked enzyme-linked fluorescence assays (ELFA) intended as an aid in the diagnosis of patients with clinical symptoms consistent with CHIKV infection. VIDAS^®^ anti-CHIKV IgM and IgG assays are qualitative immunocapture assays detecting CHIKV-specific IgM and IgG antibodies, respectively. They can be tested in parallel or independently, are rapid (40 min to result), easy to use, and easy to interpret (positive or negative) with no equivocal zone. The performance of these automated assays has not yet been directly compared to that of conventional manual assays. The aim of this international study was to evaluate for the first time the clinical performance of the VIDAS^®^ anti-CHIKV IgM and IgG assays in samples from patients with a suspected CHIKV infection, enrolled from multiple CHIK-endemic regions of the world (Asia, Latin America). The clinical performance of the VIDAS^®^ anti-CHIKV assays was compared to that of existing manual competitor ELISA and RDT assays.

## 2. Materials and Methods

### 2.1. Patients and Samples

A total of 660 sera were collected at three sites in patients with a suspected CHIKV infection and from several CHIKV-endemic regions, including Asia (India) and Latin America (Brazil, Colombia, Dominican Republic, Honduras, Peru) (Table 1). Retrospective and prospective cohorts of samples collected between January 2016 and September 2021 were used for this study. For retrospective biobanked samples, a suspicion of CHIKV infection was established based on the documented presence of one or more of the following symptoms at the time of sampling: fever, joint pain or arthritis, tenosynovitis, bursitis, headache, back pain, rash, myalgia, cutaneous pruritus, polyadenopathy, oedema of the face and extremities. For prospective samples, a suspicion of CHIKV infection was established during a routine medical procedure based on the presence of fever and joint pain or arthritis within 3 months of sampling, associated with one or more of the following symptoms: headache, back pain, rash, myalgia, cutaneous pruritus, polyadenopathy, oedema of the face and extremities.

All collected sera (≥1.0 to 1.5 mL) were aliquoted to allow testing with the different assays on the same freeze/thaw cycle. Aliquots were stored frozen at −80 °C until testing. When applicable (collection sites 2 and 3), frozen samples or aliquots were transported to the testing site under controlled conditions.

Samples were tested at two central laboratories: the Tropical Medicine Institute of the University of São Paulo, Brazil for retrospective longitudinal samples collected in Brazil (site 1; Table 1), and the Clinical Affairs Laboratory of bioMérieux, Marcy l’Etoile, France for samples collected prospectively in India (site 2; Table 1) and for retrospective biobanked samples collected in Latin America and purchased from commercial providers (site 3; Table 1).

This study was conducted in adherence to the Declaration of Helsinki and approved by the institutional ethics committee (CEP) of the Faculty of Medicine of the University of São Paulo, Brazil (approval number 4.692.542, dated 5 May 2021), and by the independent ethics committee Namaste Integrated Services, Lanka, Varanasi, India (approval number BS-IND-001, dated 7 August 2021). Purchased samples were collected and approved for use for research purposes by the respective commercial providers (Boca Biolistics, Pompano Beach, FL, USA; Trans-Hit Bio/Azenta Life Sciences, Burlington, MA, USA; ABO Pharmaceuticals, San Diego, CA, USA). All participants, or a parent or legal guardian in the case of children, provided informed consent before the start of the study.

Precision experiments were conducted using characterised negative and positive samples (bioMérieux collection). Negative samples were provided by the French blood bank (Etablissement Français du Sang [EFS], La Plaine Saint-Denis, France). Each volunteer donor signed a written informed consent form for the use of blood for research purposes. EFS obtained authorisation from the French Ministry of Research to collect and transfer samples to partners (Ministère de l’Enseignement Supérieur, de la Recherche et de l’Innovation, reference AC-2017-2958).

Cross-reactivity experiments were performed using native samples collected from patients with other potentially interfering infections who tested positive for antibodies against the respective pathogens (bioMérieux collection).

### 2.2. Study Design and Definitions

The aim of this performance evaluation study was to compare the performance of the automated VIDAS^®^ anti-CHIKV IgM and IgG assays with that of manual competitor ELISA for the detection of anti-CHIKV IgM and IgG antibodies in patients with a suspected CHIKV infection.

**Figure 1:**
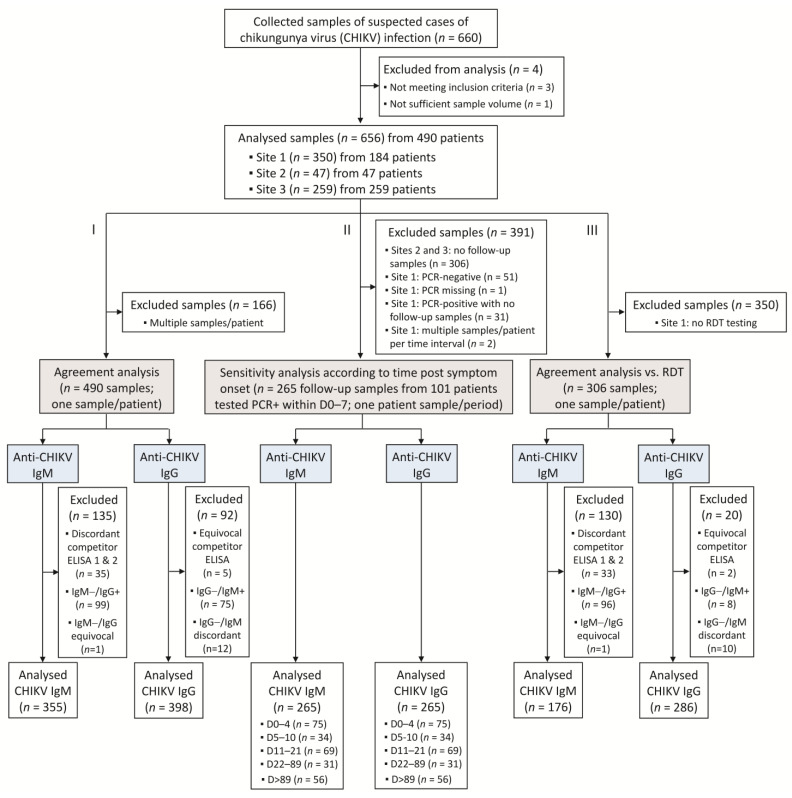
Study flow diagram. Three analyses were conducted: (**I**) an agreement analysis assessed the performance of the VIDAS^®^ CHIKV IgM and IgG assays in comparison to commercial competitor ELISA; (**II**) the sensitivity of the VIDAS^®^ CHIKV IgM and IgG assays was evaluated in patients with a confirmed CHIKV infection (defined as an rRT-PCR-positive within 7 days of symptom onset); (**III**) the agreements of VIDAS^®^ CHIKV IgM and IgG assays or of RDT IgM/IgG with competitor ELISA were evaluated on a common set of samples and compared to each other.

Three distinct analyses were performed. First, an agreement analysis was conducted on the whole cohort, comparing the results of the VIDAS^®^ CHIKV IgM and IgG assays to those of competitor ELISA, which was used as a comparative method (Table 2). To consolidate the detection of anti-CHIKV IgM antibodies, two competitor IgM ELISA methods were used (Table 2). This choice was motivated by the acknowledged non-negligible rate of false-positive and false-negative results of IgM serology assays in general [25,26], and of CHIKV IgM serology assays in particular [23,27]. To limit the bias that could be introduced in the agreement analysis by false-positive and/or false-negative results of the comparative method, the results of two well-validated commercial IgM ELISAs were taken into consideration. An IgM result by the competitor ELISA was defined as positive when both IgM ELISA tests were positive, and negative when both IgM ELISA tests were negative (Table 3). Discordant results were excluded from the analysis (Figure 1).

Positive percent agreement (PPA) analyses for anti-CHIKV IgM assays were conducted on samples positive with the competitor IgM ELISA (regardless of the IgG status). Similarly, PPA analyses for anti-CHIKV IgG assays were conducted on samples positive with the competitor IgG ELISA (regardless of the IgM status) (Table 3). For a more robust negative agreement (NPA) analysis, only samples negative for both IgM and IgG (with competitor ELISA) were included in the test comparison (Table 3). Only one sample per patient was included in the agreement analysis. In the case of multiple samples per patient, the first sample available chronologically was analysed.

This agreement analysis on the whole population was completed by an agreement analysis on the same samples but according to the time from symptom onset. The five periods investigated were defined according to the documented time intervals post symptom onset: 0–6 days, 7–10 days, 11–21 days, 22 days–3 months, and >3 months.

A second analysis was conducted on the follow-up retrospective samples collected at site 1 (Brazil; Table 1) to evaluate the sensitivity of the VIDAS^®^ anti-CHIKV IgM and IgG assays at different time points following a confirmed CHIKV infection. A CHIKV infection was defined as confirmed when positive for CHIKV RNA by rRT-PCR, set as the gold standard. Patients with a positive rRT-PCR at ≤7 days post symptom onset and at least one follow-up sample were included in this analysis (Figure 1). Five periods following symptom onset were investigated according to the documented days post symptom onset: 0–4 days, 5–10 days, 11–21 days (acute phase of CHIKV infection), 22–89 days (post-acute phase of CHIKV infection), and >89 days (chronic phase of CHIKV infection). Only one sample per patient per period was included in the analysis. In the case of multiple samples per patient per period, the first sample collected chronologically was used. The sensitivity of the VIDAS^®^ IgM and IgG assays was defined as the percentage of positive test results in patients confirmed positive for CHIKV infection.

A third analysis was conducted on backup samples from sites 2 and 3 (Table 1) to evaluate the performance of VIDAS^®^ anti-CHIKV IgM and IgG assays vs. that of an RDT (Standard Q Chikungunya IgM/IgG, SD Biosensor, Gurugram, Haryana, India). To that aim, the concordance of the VIDAS^®^ anti-CHIKV IgM and IgG assays to competitor ELISA was compared to the concordance of the RDT to the same competitor ELISA (as a comparative method). This agreement sub-analysis was conducted following the same rules as those of the agreement analysis applied to the whole cohort (see above and Table 2 and Table 3). One sample per patient was included in the analysis (Figure 1). Clinical agreement (PPA, NPA) of each method (VIDAS^®^ or RDT) with competitor ELISA was assessed independently and compared with a statistical method.

### 2.3. VIDAS^®^ Assays

VIDAS^®^ Anti-CHIKUNGUNYA IgM (CHKM; 423229) and VIDAS^®^ Anti-CHIKUNGUNYA IgG (CHKG; 423230) (bioMérieux SA, Marcy-l’Étoile, France) are automated qualitative two-step immunocapture assays combined with enzyme-linked fluorescent assay (ELFA) detection, developed for the VIDAS^®^ family of instruments. They are intended as an aid in the diagnosis of patients with clinical symptoms consistent with CHIKV infection. The Solid Phase Receptacle (SPR^®^) serves as the solid phase as well as the pipetting device. Reagents for the assay are ready-to-use and pre-dispensed in the sealed reagent strip. All steps are performed automatically by the instrument and completed within approximately 40 min. The reagents used for assay development and for this performance evaluation study are identical to those included in the commercialised CE-marked assays.

For the VIDAS^®^ Anti-CHIKUNGUNYA IgM assay (hereafter referred to as the VIDAS^®^ anti-CHIKV IgM assay), total IgM is captured by a monoclonal antibody specific for human IgM coated on the interior of the SPR. In the second step, anti-CHIKV IgM is specifically detected by a CHIKV-specific antigen and anti-CHIKV antibodies conjugated to alkaline phosphatase.

For the VIDAS^®^ Anti-CHIKUNGUNYA IgG assay (hereafter referred to as the VIDAS^®^ anti-CHIKV IgG assay), anti-CHIKV IgG is captured by the CHIKV-specific antigen coated on the interior of the SPR. In the second step, the captured anti-CHIKV IgG is detected by an antibody specific for human IgG conjugated to alkaline phosphatase.

The CHIKV-specific antigen used in both VIDAS^®^ anti-CHIKV assays is a virus-like particle (VLP) produced by transient transfection of HEK293 cells with a eukaryotic expression plasmid encoding the CHIKV capsid and envelope structural polyproteins C-E3-E2-6K-E1 (from strain 37997 of the West African lineage) [28,29]. CHIKV VLPs are composed of 240 copies of capsid proteins surrounded by the host cell plasma membrane and an outermost layer of 240 heterodimers of the immunogenic envelope proteins E1-E2, assembled into 80 glycoprotein spikes [28,29]. CHIKV-specific VLPs secreted in the culture medium were purified by ion exchange chromatography and on a multimodal resin using proprietary protocols.

During the final detection step of both VIDAS^®^ anti-CHIKV immunoassays, the substrate (4-Methyl-umbelliferyl phosphate) is cycled in and out of the SPR. The conjugate enzyme catalyzes the hydrolysis of the substrate into a fluorescent product (4-Methyl-umbelliferone). Fluorescence is measured at 450 nm and a relative fluorescence value (RFV) is generated (background reading subtracted from the final fluorescence reading). The results are automatically calculated by the instrument, according to a standard (S1), and an index value (i) is obtained (where i = RFV_sample_/RFV_S1_). The test is interpreted as negative when i < 1.0 and positive when i ≥ 1.0. The positivity cut-off values for the VIDAS^®^ CHIKM and CHKG assays were determined based on the area under the receiver operating characteristic (ROC) curve and Youden index analyses, using clinically characterised positive and negative human samples.

For the study, VIDAS^®^ anti-CHIKV IgM and IgG assays were performed and interpreted according to the instructions for use (056847-01 and 055960-01, respectively). VIDAS^®^ assays were repeated in the event of invalid calibration, established human error, or absence of results delivered by the device. Only valid repeated results were taken into account for data analysis. Two lots of VIDAS^®^ anti-CHIKV IgM and IgG assays were used, and the same lots were used at both testing sites (Brazil and France; Table 1). At the testing site in Brazil (Table 1), samples were evaluated on one VIDAS^®^ instrument and in parallel by ELISA on a Mustikan FC reader (ThermoFisher Scientific, Waltham, MA, USA) between 4 October 2021, and 18 October 2021. At the testing site in France (Table 1), two VIDAS instruments were employed, one for the VIDAS^®^ anti-CHIKV IgM assays and one for the VIDAS^®^ anti-CHIKV IgG assays. Samples were evaluated in parallel on VIDAS^®^ and by ELISA on an ELISA reader BioTek 800TS (Agilent, Santa Clara, CA, USA) between 26 July 2021, and 7 October 2021.

### 2.4. Competitor Enzyme-Linked Immunosorbent Assays (ELISAs)

Competitor ELISAs (Table 2) were conducted and interpreted according to the manufacturers’ recommendations. Competitor ELISA tests were repeated in the event of established human error or in the absence of results delivered by the ELISA reader. Only valid repeated results were taken into account for data analysis.

IgM ELISA (InBios) was interpreted as negative for result values (Immune Status Ratio [ISR]) < 0.9, positive for ISR > 1.1, and equivocal for ISR of 0.9–1.1. IgM ELISA (NovaTec) was interpreted as negative for result values (NovaTec Units [NTU]) < 9, positive for NTU > 11, and equivocal for NTU of 9–11. Equivocal IgM assays were repeated in duplicate (inBios) or singlicate (NovaTec). The repeated result (mean of duplicate for InBios, singlicate value for NovaTec) was interpreted as either negative (<1.0 for InBios, ≤11 for NovaTec) or positive (≥1.0 for InBios, >11 for NovaTec). Thus, the final interpretation of IgM competitor ELISA was either negative or positive. Discordant IgM ELISA test results were excluded from the analysis (Figure 1).

IgG ELISA (Euroimmun) was interpreted as negative for result values (Ratio) < 0.8, positive for a ratio ≥ 1.1, and equivocal for a ratio of 0.8 to <1.1. Equivocal IgG ELISA test results were excluded from the analysis (Figure 1).

### 2.5. Rapid Diagnostic Test (RDT)

The Standard Q Chikungunya IgM/IgG Rapid Kit (SD Biosensor, Gurugram, Haryana, India) was applied to backup samples of sites 2 and 3 (Table 1). The test was performed and interpreted according to the manufacturer’s instructions. In case of an invalid RDT result, the test was repeated. In the event of a repeated invalid test result, the test was confirmed as invalid and excluded from the analysis. Only valid repeated results were taken into account for data analysis.

### 2.6. Real-Time RT-PCR Assays

At the collection and testing site in Brazil (Table 1), rRT-PCR was performed on samples with a time from symptom onset ≤ 7 days using the ZDC kit (Zika, dengue, and chikungunya) from Bio-Manguinhos, a unit of Fiocruz (Institute of Technology in Immunobiologicals) approved by the National Agency for Health Surveillance ANVISA (register number 80142170032). Samples with a positive rRT-PCR result and with at least one follow-up sample were included in the sensitivity analysis.

At the testing site in France for samples collected at sites 2 and 3 (Table 1), rRT-PCR was performed on samples with a time from symptom onset ≤ 14 days for information purposes only, using the CE-approved RealStar Chikungunya RT-PCR Kit 2.0 (Altona diagnostics GmbH, Hamburg, Germany). The testing was outsourced to BIOMEX GmbH (Heidelberg, Germany).

### 2.7. Precision Experiments

Precision experiments were conducted at bioMérieux (Marcy l’Etoile, France). Assay precision was evaluated according to the CLSI EP05-A3 guideline [30] using characterised high negative, low positive, and moderate positive human serum samples, as determined by VIDAS^®^ anti-CHIKV IgM and IgG assays. Samples were prepared from negative native EFS samples spiked with a high positive native sample to obtain the expected index value levels. Samples were stored at −20 °C/−30 °C until use.

Within-run precision (repeatability) and within-laboratory precision (between-lot reproducibility) of the VIDAS^®^ anti-CHIKV IgM and IgG assays were determined on samples run in triplicate twice a day for 10 days (equivalent to a 20-day precision), using two lots of VIDAS^®^ assays on one VIDAS^®^ instrument calibrated every second day, thus generating 120 measurements per sample. A visual data integrity check was performed to identify possible outliers. Visually discordant results were confirmed to be statistical outliers using the Generalized Extreme Studentized Deviate (ESD) test with a 1% α risk. In case of confirmed outliers, data analysis was performed on both the full dataset and on the dataset without statistical outliers. Only statistical outliers with an impact on the precision estimates were considered outliers. Variance was expressed as standard deviation (SD) and coefficient of variation (CV).

### 2.8. Cross-Reactivity Experiments

The analytical specificity of the VIDAS^®^ anti-CHIKV IgM and IgG assays was evaluated at bioMérieux (Marcy l’Etoile, France) on samples containing potentially interfering antibodies directed against other pathogens. Cross-reactivity experiments were performed using native samples collected from patients who tested positive for antibodies against related or unrelated pathogens, as follows. Samples used for evaluating the cross-reactivity with VIDAS^®^ anti-CHIKV IgM were positive for pathogen-specific IgM, except for HAV, HBV, HCV, HIV, IAV/IBV, and *Plasmodium falciparum* samples, which were positive for pathogen-specific total antibodies. Samples used for evaluating the cross-reactivity with anti-CHIKV IgG were positive for pathogen-specific IgG, except for HAV, HBV, HCV, HIV, IAV/IBV, YFV, and *Plasmodium falciparum* samples, which were positive for pathogen-specific total antibodies.

In addition, samples tested with VIDAS^®^ anti-CHIKV IgM were previously characterized as negative using a competitor anti-CHIKV IgM ELISA (Euroimmun Anti-Chikungunya Virus ELISA (IgM), Inbios CHIKjj Detect™ IgM ELISA or NovaLisa^®^ Chikungunya Virus IgM μ-capture). Samples tested with VIDAS^®^ anti-CHIKV IgG were previously characterized as negative using the Euroimmun Anti-Chikungunya Virus ELISA (IgG).

All samples were stored at −80 °C until use, except for samples of SARS-CoV-2-infected patients, which were stored at −30 °C. Samples were tested in singlicate, using one kit lot each (IgM, IgG) on either five VIDAS^®^ instruments (IgM) or two VIDAS^®^ instruments (IgG). A total of 210 and 205 samples with other potentially interfering infections were tested on the VIDAS^®^ anti-CHIKV IgM and IgG assays, respectively.

### 2.9. Statistical Analyses

Agreement analyses were conducted between the VIDAS^®^ assays and competitor ELISA used as a comparative method. Agreement analyses (PPA, NPA, and overall percent agreement) were performed in adherence to the CLSI EP12-A2 guideline [31]. The 95% confidence intervals (95% CI) were computed, either as Wilson Score Confidence Interval if the percentage agreement was in the range ]5%, 95%[ or as Exact Binomial Confidence Interval otherwise, using the SAS Enterprise Guide 7.12 software.

The sensitivity of the VIDAS^®^ IgM and IgG assays was evaluated by determining the percentage of positive VIDAS^®^ results on follow-up samples of patients with a CHIKV rRT-PCR-positive status established between day 0 and 7 post symptom onset. The respective 95% CIs were computed as above. The sensitivity of the competitor IgM and IgG ELISA was evaluated in parallel and compared to that of the VIDAS^®^ assays in a pairwise comparison using a McNemar’s test with Bonferroni correction (correction for three tests for IgM assays, and for two tests for IgG assays).

Agreement of the VIDAS^®^ and RDT assays with competitor ELISA was compared according to the CLSI EP12-A2 guidelines [31], using the 95% CI of the differences of these two concordance values; if 0 belonged to the 95% CI then both concordance values were not considered significantly different, while if 0 was outside the 95% CI then both concordance values were considered significantly different.

The assay precision was assessed in adherence to the CLSI EP05-A3 guideline [30] by a component-of-variance analysis for nested design (Restricted Maximum Likelihood) using the SAS Enterprise Guide 7.12 software.

VIDAS^®^ CHIK IgM and IgG index values of longitudinal study samples used for the sensitivity analysis (i.e., in patients with a confirmed CHIKV infection) were displayed as Tukey box plots according to the time post symptom onset, using GraphPad Prism 5.04 (GraphPad Software, San Diego, CA, USA).

## 3. Results

### 3.1. Patients’ Characteristics

A total of 660 serum samples were collected, of which 656 were analysed (Figure 1). The 656 included samples were from 490 patients with suspected CHIKV infection, as described in Table 4. The whole study population was composed of 340 (69.4%) females and presented a median (range) age of 37 (15–92) years (Table 4). Out of the 490 included patients, 184 (37.5%) were from Brazil, 165 (33.7%) from Colombia, 72 (14.7%) from Peru, 47 (9.6%) from India, 16 (3.3%) from the Dominican Republic, and 6 (1.2%) from Honduras (Table 4).

A total of 490 samples were included in the agreement analysis comparing the VIDAS^®^ anti-CHIKV IgM and IgG assays to competitor ELISA (Figure 1, analysis I), 265 follow-up samples of patients confirmed positive for CHIKV infection were included in the sensitivity analysis (Table S1 and Figure 1, analysis II), and 306 samples were part of the agreement sub-analysis comparing VIDAS^®^ assays to ELISA vs. RDT to ELISA (Figure 1, analysis III).

### 3.2. Clinical Performance of the VIDAS^®^ Anti-CHIKV IgM and IgG Assays

#### 3.2.1. Clinical Sensitivity

The sensitivity of the VIDAS^®^ assays was evaluated in patients confirmed positive for CHIKV infection (as determined by a positive CHIKV rRT-PCR at ≤7 days post symptom onset; Table S1). Clinical sensitivity was defined as the percentage of positive test results and was evaluated at different time intervals following the onset of symptoms. Sensitivity of the competitor ELISA was evaluated in parallel (Table 5 and Figure S1).

All evaluated anti-CHIKV IgM assays demonstrated high sensitivity (88.2–100.0%) from day 5 post symptom onset, while all anti-CHIKV IgG assays showed 100.0% sensitivity from day 11 post symptom onset (Table 5 and Figure S1). Anti-CHIKV IgM and IgG assays presented a lower sensitivity at earlier time points after the onset of symptoms (≤24.0% for CHIKV IgM assays at 0–4 days, and ≤26.5% for CHIKV IgG assays at 0–10 days; Table 5 and Figure S1), as predicted from the reported kinetics of antibody response following a CHIKV infection [5,6,19,20].

Altogether, over the whole evaluated period, few differences in sensitivity were observed between the compared assays, with differences of 1.9% (5/265) between VIDAS^®^ CHIKV IgM and inBios IgM ELISA, 4.5% (12/265) between VIDAS^®^ CHIKV IgM and NovaTec IGM ELISA, and 1.5% (4/265) between VIDAS^®^ CHIKV IgG and Euroimmun IgG ELISA (Table 5). Pairwise differences in sensitivity were evaluated in case of apparent differences in proportions at earlier time points (0–4 days for IgM assays, 0–4 days and 5–10 days for IgG assays; Table 5) using an exact McNemar’s test with Bonferroni correction. All pairwise differences in sensitivity were not statistically significant (*p* = 1.000 for VIDAS^®^ CHIKV IgM vs. InBios IgM ELISA at 0–4 days, *p* = 0.094 for NovaTec IgM ELISA vs. InBios IgM ELISA at 0–4 days, *p* = 1.000 for VIDAS^®^ CHIKV IgG vs. Euroimmun IgG ELISA at both 0–4 and 5–10 days), except for the comparison of VIDAS^®^ CHIKV IgM vs. NovaTec IgM ELISA at 0–4 days (*p* = 0.047). However, a closer evaluation of the result values of the 12 apparent discordant VIDAS^®^ CHIKV IgM test results vs. NovaTec IgM ELISA (corresponding to samples negative for VIDAS^®^ and positive for NovaTec ELISA; Table 5) revealed low positive test results for the NovaTec IgM ELISA (with a median [IQR] of 14.4 [13.6–14.9], close to the positivity cutoff of 11.0), indicating no major discordance between test results, and thus no great differences in sensitivity.

**Figure 2:**
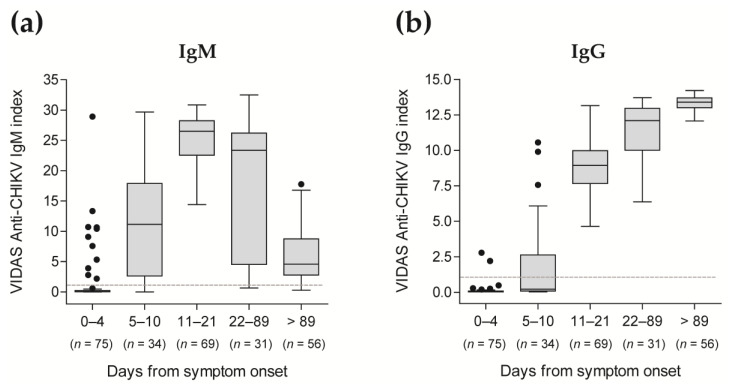
Distribution of VIDAS^®^ CHIKV IgM (**a**) and IgG (**b**) index values in patients confirmed positive for CHIKV infection according to the time post symptom onset. VIDAS^®^ CHIKV IgM and IgG index values of 265 samples from 101 CHIKV-positive patients (as determined by rRT-PCR within 7 days of symptom onset) are displayed as Tukey box plots according to the time from symptom onset. No more than one patient’s sample is included per period. The dashed horizontal line shows the positivity cut-off of both assays (i = 1.0). The median and interquartile range of the respective index values are shown in Table S2.

In agreement with these qualitative test results, index values of the VIDAS^®^ CHIKV IgM and IgG assays showed the expected kinetics of the antibody response [5,6,19,20], with significant detection of anti-CHIKV IgM from day 5 after onset of symptoms, peaking at 11–21 days, subsiding afterward (Figure 2a and Table S2), while anti-CHIKV IgG strongly increased from day 11 after onset of symptoms and remained high over the evaluated period (Figure 2b and Table S2).

#### 3.2.2. Concordance of the VIDAS^®^ CHIKV IgM and IgG Assays with Competitor ELISA in the Total Study Population

Agreement analysis comparing the performance of the VIDAS^®^ CHIKV IgM and IgG assays to that of the competitor ELISA demonstrated very high positive and negative percent agreements (PPA and NPA between 97.5% and 100.0%; Table 6). The PPA (95% CI) of the comparison of anti-CHIKV IgM assays was the lowest, with 97.5% (93.8–99.3%).

Altogether, very few test results were discordant between the VIDAS^®^ CHIKV assays and the comparative methods, with 4/355 (1.1%) discordant anti-CHIKV IgM assays and 2/398 (0.5%) anti-CHIKV IgG assays (Table 6). For IgM assays, evaluation of the four discordant samples (negative for VIDAS^®^ CHIKV IgM and positive for the competitor IgM ELISA; Table 6) revealed inBios IgM test results close to the positivity cutoff of 1.0 and NovaTec IgM test results that were moderately positive (median [IQR] of 17.95 [14.96–21.81]). These four discordant samples were collected early after symptom onset (0–6 days; Table S3). For IgG assays, the two discordant samples (one in the PPA analysis and one in the NPA analysis; Table 6) revealed test results close to the respective positivity cutoff, thus not strongly discordant. As for IgM assays, the one discordant PPA result (negative for VIDAS^®^ CHIKV IgG and positive for the competitor IgG ELISA) was from a sample collected within 0–6 days of symptom onset (Table S3), when the antibody response starts to mount (see Figure 2). The one discordant NPA result in the anti-CHIKV IgG assay comparison (positive for VIDAS^®^ CHIKV IgG and negative for the competitor IgG ELISA) was collected in the post-acute phase (22 days–3 months post symptom onset; Table S3).

#### 3.2.3. Comparison of Assay Concordance of the VIDAS^®^ CHIKV Assays and RDT with Competitor ELISA

An agreement sub-analysis was conducted aiming to compare on common samples the agreement of VIDAS^®^ assays with the competitor ELISA to that of lateral flow RDT with the same competitor ELISA (Figure 1). In this sub-cohort, VIDAS^®^ CHIKV IgM and IgG assays showed PPA and NPA with the competitor ELISA close to 100.0% (ranging from 99.2% to 100.0%; Table 7). By contrast, the PPA of RDT IgM/IgG with the competitor ELISA was moderate (68.4% and 67.4% for IgM and IgG, respectively), together with an NPA of 100.0% (Table 7).

Differences in agreement to ELISA of the VIDAS^®^ and RDT assays were tested using the 95% CI of the differences of both concordance values, as described in the Materials and Methods (Section 2.9). For anti-CHIKV IgM assays, the NPA of VIDAS^®^ and RDT assays were both in perfect concordance with the comparative method (100.0%; Table 7). By contrast, the difference (95% CI) of the PPA of the VIDAS^®^ and RDT assays was 31.6% (7.61–53.99), indicating that the PPA of VIDAS^®^ CHIKV IgM to ELISA was significantly higher than that of the RDT IgM. Similarly, for anti-CHIKV IgG assays, the difference (95% CI) of the NPA of the VIDAS^®^ and RDT assays was −0.6% (−3.50–1.77), inferring that the NPA of VIDAS^®^ CHIKV IgG to ELISA was not significantly different from that of RDT IgG. As for IgM assays, the PPA of VIDAS^®^ CHIKV IgG (99.2%) was significantly higher than that of the RDT IgG (67.4%), with a difference (95% CI) in the PPA of VIDAS^®^ and RDT assays of 31.8% (23.54–40.31). Therefore, while the NPA of the VIDAS^®^ anti-CHIKV IgM and IgG assays were comparable to that of the rapid test STANDARD^TM^ Q IgM/IgG (both close to 100.0%), the PPA of VIDAS^®^ anti-CHIKV IgM and IgG assays were significantly higher than that of the RDT.

A closer evaluation of the discordant RDT test results in the PPA analysis (i.e., negative for RDT and positive for the competitor ELISA) showed that the 6/19 (31.6%) samples with negative RDT IgM results (Table 7) were moderately positive with the NovaTec IgM ELISA (median NTU of 24.6), the inBios IgM ELISA (median ISR of 7.7), and the VIDAS^®^ CHIKV IgM (median index of 14.3). Similarly, 42/129 (32.6%) samples with negative RDT IgG results (Table 7) were moderately positive with the Euroimmun IgG ELISA (median ratio of 4.28), and 41/42 of those were moderately positive with the VIDAS^®^ CHIKV IgG (median index of 14.2). Altogether, these results demonstrate that the RDT IgM/IgG assay is less sensitive than ELISA, but also less sensitive than the VIDAS^®^ CHIKV assays.

### 3.3. Analytical Performance of the VIDAS^®^ CHIKV IgM and IgG Assays

#### 3.3.1. Assay Precision

The assay precision of the VIDAS^®^ anti-CHIKV IgM and IgG assays was evaluated on high negative, low positive, and moderate positive samples. No outliers were identified and a total of 120 measurements were included in the precision calculation. The coefficient of variation (CV) across both assays did not exceed 5.0% for repeatability (within-run precision) and 7.4% for within-laboratory (between-lot) precision (Table 8).

#### 3.3.2. Assay Cross-Reactivity

The analytical specificity of the VIDAS^®^ anti-CHIKV IgM and IgG assays was evaluated using samples from patients with other proven infections and who had tested positive for the respective pathogen-specific IgM, IgG, or total antibodies, and tested negative with the respective competitor ELISA. The potentially interfering pathogens evaluated were those responsible for febrile infections that could be misdiagnosed as CHIKV, such as other alphaviruses (Barmah Forest virus, Ross River virus), flaviviruses (dengue virus, West Nile virus, yellow fever virus, Zika virus, Japanese encephalitis virus), or other pathogens (*Plasmodium falciparum*, leptospira, severe acute respiratory syndrome coronavirus 2 [SARS-CoV-2], …). Cross-reactivity was measured as the proportion of positive VIDAS^®^ anti-CHIKV IgM and IgG test results among these samples (Table 9).

Overall cross-reactivity with the VIDAS^®^ anti-CHIKV IgM and IgG assays was very low (1/210 [0.5%] for VIDAS^®^ anti-CHIKV IgM and 6/205 [2.9%] for VIDAS^®^ anti-CHIKV IgG; Table 9). The VIDAS^®^ anti-CHIKV IgM assay showed one cross-reactivity with a native sample positive for *Toxoplasma gondii*-specific IgM. The VIDAS^®^ anti-CHIKV IgG assay was cross-reactive with one native sample positive for herpes simplex virus (HSV)-specific IgG, and with five samples positive for IgG against mosquito-borne viruses, including West Nile virus (two cross-reactive samples out of ten tested) and Ross River virus (three cross-reactive samples out of ten tested). No VIDAS^®^ anti-CHIKV IgG cross-reactivity was observed with IgG-positive samples of patients infected with other flaviviruses such as dengue, yellow fever, and Zika viruses. Similarly, the VIDAS^®^ anti-CHIKV IgM assay showed no cross-reactivity with samples of patients who tested IgM-positive for any of the investigated alphaviruses (Barmah Forest virus, Ross River virus) and flaviviruses (dengue virus, West Nile virus, yellow fever virus, Zika virus, Japanese encephalitis virus). Moreover, VIDAS^®^ anti-CHIKV IgM and IgG assays demonstrated no cross-reactivity with samples positive for SARS-CoV-2 IgM and IgG antibodies, respectively (Table 9).

## 4. Discussion

This international study assessed the clinical performance of the automated VIDAS^®^ anti-CHIKV IgM and IgG assays in comparison to a manual competitor ELISA used as a comparative method and evaluated assay sensitivity in patients with a confirmed CHIKV infection.

In this first performance evaluation study, VIDAS^®^ anti-CHIKV IgM and IgG results were comparable to those of competitor IgM and IgG ELISAs, with positive and negative agreements between 97.5% and 100.0%. Given that existing commercial anti-CHIKV IgM and IgG ELISA are recognised for their ability to accurately detect anti-CHIKV antibodies [5,6,10,11,19,20,22,23,24], our study, therefore, demonstrates the good clinical performance of the VIDAS^®^ anti-CHIKV IgM and IgG assays. Our study also confirmed the superior performance of ELISA over RDT, in accordance with the existing literature [5,6,10,11,19,20,22,23,24]. Moreover, the significant difference between the PPA of VIDAS^®^ and RDT assays (each compared to competitor ELISA) demonstrates for the first time the superior performance of VIDAS^®^ anti-CHIKV assays over that of RDT. On the other hand, both VIDAS^®^ and RDT assays showed NPA near or equal to 100.0%, suggesting a clinical specificity comparable to that of ELISA.

Sensitivity of the VIDAS^®^ anti-CHIKV IgM and IgG assays, assessed as the percentage of positive test results in patients with a proven CHIKV infection, confirmed reports from the literature as to the kinetics of the anti-CHIKV antibody response [5,6,19,20], with >88% IgM detection at ≥5 days and 100% IgG detection at ≥11 days after symptom onset. Our results, therefore, support the current guidelines for CHIKV infection diagnosis, recommending the detection of CHIKV RNA by real-time RT-PCR within the first week of symptom onset, and detection of anti-CHIKV IgM and/or IgG thereafter [13,15,17,18].

In addition to their strong clinical performance, VIDAS^®^ anti-CHIKV IgM and IgG assays demonstrated excellent analytical performance with high precision (CV < 8%) and analytical specificity (cross-reactivities < 3%). Few cross-reactivities were identified using samples of patients with related or unrelated infections. Nonetheless, five samples that were positive for IgG against mosquito-borne arboviruses (2/10 West Nile virus and 3/10 Ross River virus), but negative with the competitor IgG ELISA, were positive with the VIDAS^®^ anti-CHIKV IgG assay. Cross-reactivity with samples of patients with a past alphavirus infection, such as Ross River virus, was expected given the close homology of alphaviruses [32,33,34] and previous reports of immune cross-reactivities between sera of patients infected with CHIKV and other alphaviruses, including O’nyong-nyong, Mayaro, and Ross River viruses [27,34,35,36,37]. These potentially cross-reactive viruses, together with CHIKV, are endemic in partly overlapping regions and are responsible for diseases presenting similar symptoms [1,38,39]. This emphasizes the potential risk of misdiagnosis, even with good-performing assays, and the importance of conducting differential diagnosis and combining rRT-PCR, IgM, and/or IgG testing, depending on the time after symptom onset, to confirm a CHIKV infection, as recommended [13,15,17,18].

The major strengths of this study include the enrolment of a large number of samples (*n* = 660) covering multiple endemic regions of the world, including Asia (India) and Latin America (Brazil, Colombia, Peru, Dominican Republic, Honduras), the large number of samples included in the cross-reactivity analysis, the parallel evaluation of samples with VIDAS^®^ and competitor assays, and the use of a unique rRT-PCR test in one central lab for the confirmation of a CHIKV infection for the sensitivity analysis.

Our study presents, nevertheless, a few limitations. First, the choice of considering two competitor IgM ELISAs as comparators to the VIDAS^®^ anti-CHIKV IgM assay led to the exclusion of 35 samples (out of 490 [7.1%]) from the IgM analysis (because of discordance between the two competitor IgM ELISAs), which might have introduced a bias in the analysis. In the absence of a gold standard anti-CHIKV IgM assay, this strategy, however, allowed a more robust agreement analysis of the VIDAS^®^ anti-CHIKV IgM assay. A second possible limitation is the selection of samples negative for both IgM and IgG (with competitor ELISAs) for NPA analyses, which led to the exclusion of 187 ‘mismatched’ IgM/IgG samples and might have introduced a bias in the analysis. This is, however, unlikely, since an analysis including all samples yielded comparable results. Third, given the heterogeneity in the number of recruited samples per site (ranging from 47 to 350; Table 4), no analysis per site was conducted. However, a preliminary analysis indicated comparable performance of the VIDAS^®^ anti-CHIKV assays per site and in the pooled cohort. A future multicentre study enrolling sufficient participants per site should fill this gap. Finally, although we tested the potential cross-reactivity of the VIDAS^®^ anti-CHIKV assays with some alphaviruses (Ross River virus, Barmah Forest virus), the difficulty to acquire alphavirus-specific sera prevented us from testing further cross-reactivities with other related alphaviruses, notably O’nyong-nyong and Mayaro viruses, known to cross-react in competitor ELISA assays [27,34,35,36,37]. Additional investigations will be needed to address this question.

## 5. Conclusions

This international performance evaluation study, conducted on a large number of samples representative of several chikungunya-endemic regions, demonstrated the excellent analytical and clinical performances of the VIDAS^®^ anti-CHIKV assays for the detection of CHIKV-specific IgM and IgG following CHIKV infection. The VIDAS^®^ anti-CHIKV assays, therefore, fulfil the requirements of the current guidelines for the diagnosis of a CHIKV infection. Furthermore, they present the advantage over conventional ELISA to be executed and interpreted automatically within 40 min, which is a clear clinical and epidemiological benefit in CHIKV endemic regions and at the time of outbreaks. They also offer more testing flexibility over ELISA (single testing vs. batch testing), and are as easy to perform as RDT, while offering a higher clinical performance than these rapid tests.

## Figures and Tables

**Table 1:** Study samples.

Site	Collection Site	Samples	Collection Time	Testing Site
**1**	National Institute of Infectious Diseases-Fiocruz, Rio de Janeiro, Brazil	Retrospective, follow-up cohort	18 April 2019–1 November 2019	Tropical Medicine Institute, Faculty of Medicine of the University of São Paulo, Brazil
**2**	Biospecimen Solutions Pvt Ltd., Sampigehalli, Bangalore, Karnataka, India	Prospective cohort	24 July 2021– 20 September 2021	Clinical Affairs Laboratory, bioMérieux, Marcy l’Etoile, France
**3**	Colombia, Dominican Republic, Honduras, and Peru	Retrospective cohort ^1^	28 January 2016–8 September 2020	Clinical Affairs Laboratory, bioMérieux, Marcy l’Etoile, France

^1^ Samples purchased from Boca Biolistics (Pompano Beach, FL, USA), Trans-Hit Bio/Azenta Life Sciences (Chelmsford, MA, USA), and ABO Pharmaceuticals (San Diego, CA, USA).

**Table 2:** Competitor ELISA used for the agreement analysis with the VIDAS^®^ CHIKV IgM and IgG assays.

Competitor ELISA	Name of Assay	Manufacturer
**IgM ^1^**	CHIKjj Detect^TM^ IgM ELISA (CHKM-R)	InBios International, Seattle, WA, USA
	NovaLisa Chikungunya Virus IgM µ-capture, ELISA Kit (NOVCHIM0590)	NovaTec Immundiagnostica, Dietzenbach, Germany
**IgG**	Chikungunya virus (CHIKV) (EI 293a-9601 G)	Euroimmun, Lübeck, Germany

^1^ The results of both IgM ELISAs were used to interpret the competitor IgM ELISA results (considered positive when both were positive, negative when both were negative, and undetermined when at least one was discordant). Sensitivity and specificity of the respective competitor ELISA, as reported in the package insert by the respective manufacturers, were: 100% sensitivity and specificity for both IgM ELISA, >96.8% sensitivity and 98.0% specificity for the IgG ELISA.

**Table 3:** Definitions of samples used for agreement analysis (PPA, NPA) according to results of competitor ELISA.

Sample Definition	IgM Competitor ELISA Results ^1^		IgG Competitor ELISA Result ^2^	CHIKV IgM Agreement Study	CHIKV IgG Agreement Study
	InBios	NovaTec	Euroimmun		
**IgM+/IgG−**	positive	positive	negative	PPA	N/A
**IgM+/IgG+**	positive	positive	positive	PPA	PPA
**IgM−/IgG+**	negative	negative	positive	N/A	PPA
**IgM−/IgG−**	negative	negative	negative	NPA ^3^	NPA ^3^

^1^ Anti-CHIKV IgM competitor ELISA, as described in Table 2; both IgM assays must be concordant (negative or positive); samples with discordant competitor IgM ELISA results were excluded from the analysis (see Figure 1). ^2^ Anti-CHIKV IgG competitor ELISA, as described in Table 2. ^3^ Only samples negative for both IgM and IgG were included in the NPA analyses. Abbreviations: N/A, not applicable (samples excluded from the respective NPA analyses); NPA, negative percent agreement; PPA, positive percent agreement.

**Table 4:** Patients’ and samples’ characteristics.

Characteristics	Total	Site 1	Site 2	Site 3
Centre	-	Brazil	India	France
Study population, *N* (%)	490 (100.0%)	184 (37.5%)	47 (9.6%)	259 (52.9%) ^1^
Age in years, median (range)	37.0 (15–92)	41.0 (19–83)	43.0 (20–83)	33.0 (15–92)
Sex, *N* (%)				
Female	340 (69.4%)	124 (67.4%)	19 (40.4%)	197 (76.1%)
Male	150 (30.6%)	60 (32.6%)	28 (59.6%)	62 (23.9%)
Study samples, *N* (%)	656 (100.0%)	350 (53.3%)	47 (7.2%)	259 (39.5%)

^1^ Out of the 259 purchased samples, 165 (63.7%) were from Colombia, 72 (27.8%) from Peru, 16 (6.2%) from the Dominican Republic, and 6 (2.3%) from Honduras.

**Table 5:** Percentage of positive test results with VIDAS^®^ assays and competitor ELISA in patients confirmed positive for CHIKV infection according to the time post symptom onset (*n* = 265 samples from 101 patients; see Figure 1 and Table 4).

Assay		Time from Symptom Onset				
		Acute Phase			Post-Acute Phase	Chronic Phase
		0–4 Days (*n* = 75)	5–10 Days (*n* = 34)	11–21 Days (*n* = 69)	22–89 Days (*n* = 31)	>89 Days (*n* = 56)
VIDAS^®^ CHIKV IgM	*n*/*N* ^1^ (%)	11/75 (14.7%)	30/34 (88.2%)	69/69 (100.0%)	30/31 (96.8%)	53/56 (94.6%)
	[95% CI]	[8.4–24.4]	[73.4–95.3]	[94.8–100.0]	[83.3–99.9]	[85.4–98.2]
InBios ELISA IgM	*n*/*N* ^1^ (%)	12/75 (16.0%)	31/34 (91.2%)	69/69 (100.0%)	29/31 (93.5%)	55/56 (98.2%)
	[95% CI]	[9.4–25.9]	[77.0–97.0]	[94.8–100.0]	[79.3–98.2]	[90.4–100.0]
NovaTec ELISA IgM	*n*/*N* ^1^ (%)	18/75 (24.0%)	31/34 (91.2%)	69/69 (100.0%)	31/31 (100.0%)	56/56 (100.0%)
	[95% CI]	[15.8–34.8]	[77.0–97.0]	[94.8–100.0]	[88.8–100.0]	[93.6–100.0]
VIDAS^®^ CHIKV IgG	*n*/*N* ^1^ (%)	2/75 (2.7%)	9/34 (26.5%)	69/69 (100.0%)	31/31 (100.0%)	56/56 (100.0%)
	[95% CI]	[0.3–9.3]	[14.6–43.1]	[94.8–100.0]	[88.8–100.0]	[93.6–100.0]
Euroimmun ELISA IgG	*n*/*N* ^1^ (%)	1/75 (1.3%)	6/33 (18.2%) ^2^	69/69 (100.0%)	31/31 (100.0%)	56/56 (100.0%)
	[95% CI]	[0.0–7.2]	[8.6–34.4]	[94.8–100.0]	[88.8–100.0]	[93.6–100.0]

^1^ *n*/*N* is the ratio of the number of samples positive for the respective immunoassays to the number of rRT-PCR-positive samples. ^2^ One sample with an equivocal result with the Euroimmun IgG ELISA assay was excluded from the calculation. An exact McNemar’s test with Bonferroni correction showed a significant difference in sensitivity between the VIDAS^®^ CHIK IgM and the NovaTec ELISA IgM assays for the 0–4 days samples (*p* = 0.047). All other pairwise comparisons of the 0–4 days (IgM, IgG) and 5–10 days (IgG) samples were not statistically significant. The percentage of positive test results according to the time post symptom onset shown in this Table are depicted in Figure S1. Abbreviations: CHIK, chikungunya; CI, confidence interval.

**Table 6:** Concordance of the VIDAS^®^ CHIKV assays with the respective competitor ELISA (*n* = 355 for anti-IgM assays, *n* = 398 for anti-IgG assays; see Figure 1).

VIDAS^®^ CHIKV Assay		Positive Percent Agreement (PPA)	Negative Percent Agreement (NPA)	Overall Percent Agreement (OPA)
**IgM**	*n*/*N* ^1^ (%)	157/161 (97.5%)	194/194 (100.0%)	351/355 (98.9%)
	[95% CI]	[93.8–99.3]	[98.1–100.0]	[97.1–99.7]
**IgG**	*n*/*N* ^1^ (%)	203/204 (99.5%)	193/194 (99.5%)	396/398 (99.5%)
	[95% CI]	[97.3–100.0]	[97.2–100.0]	[98.2–99.9]

^1^ *n*/*N* is the ratio of the number of samples for which VIDAS^®^ assays are in agreement (positive, negative, and overall) with the competitor ELISA (comparative method) to the number of samples that tested either positive or negative (and overall) with the competitor ELISA. Positive and negative agreement with competitor ELISA was calculated according to the rules described in Table 3. Abbreviations: CI, confidence interval.

**Table 7:** Concordance of the VIDAS^®^ CHIKV assays and of the RDT IgM/IgG assay with competitor ELISA (*n* = 176 for IgM, *n* = 286 for IgG; see Figure 1).

Assay		Positive Percent Agreement (PPA)	Negative Percent Agreement (NPA)	Overall Percent Agreement (OPA)
**VIDAS^®^** **CHIKV** **IgM**	*n*/*N* ^1^ (%)	19/19 (100.0%)	157/157 (100.0%)	176/176 (100.0%)
	[95% CI]	[82.4–100.0]	[97.7–100.0]	[97.9–100.0]
**RDT** **IgM/IgG ^2^**	*n*/*N* ^1^ (%)	13/19 (68.4%)	157/157 (100.0%)	170/176 (96.6%)
	[95% CI]	[46.0–84.6]	[97.7–100.0]	[92.7–98.7]
**VIDAS^®^** **CHIKV** **IgG**	*n*/*N* ^1^ (%)	128/129 (99.2%)	156/157 (99.4%)	284/286 (99.3%)
	[95% CI]	[95.8–100.0]	[96.5–100.0]	[97.5–100.0]
**RDT** **IgM/IgG ^2^**	*n*/*N* ^1^ (%)	87/129 (67.4%)	157/157 (100.0%)	244/286 (85.3%)
	[95% CI]	[59.0–74.9]	[97.7–100.0]	[80.7–88.9]

^1^ *n*/*N* is the ratio of the number of samples for which VIDAS^®^ or RDT assays are in agreement (positive, negative, and overall) with the competitor ELISA (reference test) to the number of samples that tested either positive or negative (and overall) with the competitor ELISA. ^2^ Standard Q Chikungunya IgM/IgG (SD Biosensor). Positive and negative agreement with competitor ELISA was calculated according to the rules described in Table 3. Abbreviations: CI, confidence interval; RDT, rapid diagnostic test.

**Table 8:** Precision of the VIDAS^®^ CHIKV IgM and IgG assays.

VIDAS^®^ CHIKV Assay	Sample	Measurements (*N*)	Mean Index	Repeatability (Within-Run Precision)		Within-Laboratory Precision ^1^	
				SD	CV (%)	SD	CV (%)
**IgM**	High negative	120	0.84	0.02	2.2	0.04	5.1
	Low positive	120	1.39	0.03	2.5	0.06	4.6
	Moderate positive	120	3.68	0.07	1.9	0.11	3.0
**IgG**	High negative	120	0.92	0.05	5.0	0.07	7.4
	Low positive	120	1.32	0.06	4.6	0.07	5.6
	Moderate positive	120	5.82	0.22	3.7	0.34	5.9

^1^ Between-lot reproducibility. Abbreviations: SD, standard deviation; CV, coefficient of variation.

**Table 9:** Cross-reactivity of human native samples from patients with other infections potentially interfering with the VIDAS^®^ CHIKV IgM and IgG assays.

Potentially Interfering Infections	Proportion of Cross-Reactions with VIDAS^®^ CHIKV Assays	
	IgM	IgG
Herpes simplex virus (HSV1/2)	0/10	1/10
Varicella zoster virus (VZV)	0/10	0/10
Cytomegalovirus (CMV)	0/11	0/10
Epstein-Barr virus (EBV)	0/9	0/10
Influenza virus (IAV/IBV)	0/12	0/12
Hepatitis A virus (HAV)	0/10	0/10
Hepatitis B virus (HBV)	0/10	0/10
Hepatitis C Virus (HCV)	0/10	0/10
Parvovirus B19	0/6	0/10
Human immunodeficiency virus (HIV)	0/10	0/10
*Borrelia burgdorferi*	0/10	0/10
*Plasmodium falciparum*	0/10	0/10
*Toxoplasma gondii*	1/12	0/10
Leptospira	0/11	0/10
Dengue virus (DENV)	0/10	0/10
West Nile virus (WNV)	0/10	2/10
Yellow fever virus (YFV)	0/10	0/10
Zika virus (ZIKV)	0/11	0/10
Japanese encephalitis virus (JEV)	0/5	n.d.
Barmah Forest virus (BFV)	0/2	0/3
Ross River virus (RRV)	0/10	3/10
Severe acute respiratory syndrome coronavirus 2 (SARS-CoV-2)	0/11	0/10
**Total, *n*/*N* (%)**	**1/210 (0.48%)**	**6/205 (2.93%)**

Abbreviation: n.d., not determined.

## Data Availability

The data presented in this study are available within the article and Supplementary Material.

## Supplementary Materials

The following supporting information can be downloaded at: https://www.mdpi.com/article/10.3390/diagnostics13132306/s1, Table S1: Description of the follow-up samples of patients confirmed positive for CHIKV infection and included in the sensitivity analysis (Figure 1, analysis II); Table S2: Median and interquartile range (IQR) of VIDAS® CHIK IgM and IgG results depicted in Figure 2, according to the time from symptom onset (*n* = 265); Table S3: Concordance of the VIDAS® CHIKV assays with the respective competitor ELISA according to the time from symptom onset (*n* = 355 samples for anti-IgM assays, *n* = 398 samples for anti-IgG assays [Figure 1]; see Table 6 for the concordance in the whole study population); Figure S1: Sensitivity of the VIDAS and competitor ELISA CHIK IgM (a) and IgG (b) assays.
