# Supplementary material for: Performance Evaluation of VIDAS® Diagnostic Assays Detecting Anti-Chikungunya Virus IgM and IgG Antibodies: An International Study
Source: Diagnostics (Basel). 2023 Jul 7;13(13):2306. doi: 10.3390/diagnostics13132306 (PMC10340453; doi:10.3390/diagnostics13132306)
Supplement: Supplementary file 1 [file diagnostics-13-02306-s001.zip › diagnostics-2392850-supplementary.pdf]

Supplementary Materials

Table S1. Description of the follow-up samples of patients confirmed positive for CHIKV infection (RT-PCR+ at ≤ 7 days from symptom onset) and included in the sensitivity analysis (Figure 1, analysis II)

**Characteristics**	
Center	Site 1 (Brazil)
Samples of RT-PCR-positive patients, *N* [1]	267
Samples of RT-PCR-positive patients, per infection stage and time from symptom onset, *N* (%)	265 (100.0%) [2]
Acute phase (≤ 3 weeks)	
0 – 4 days	75 (28.3%)
5 – 10 days	34 (12.8%)
11 – 21 days	69 (26.1%)
Post-acute phase (> 3 weeks to 3 months)	
22 – 89 days	31 (11.7%)
Chronic phase (> 3 months)	
> 89 days [3]	56 (21.1%)

[1] Out of the 350 samples from 184 patients at site 1 (Table 4), 267 samples from 101 patients (2 to 3 samples per patient) were tested positive for CHIKV RNA by RT-PCR within 7 days of symptom onset; [2] Two out of 267 samples were excluded from the sensitivity analysis due to multiple samples per patient per period; [3] For the chronic phase, samples were collected between 90 and 106 days.

Table S2. Median and interquartile range (IQR) of VIDAS® CHIK IgM and IgG index values depicted in Figure 2, according to the time from symptom onset (n=265 samples from 101 patients; no more than one sample per time period)

**VIDAS® CHIK assay**	**Variable**	**Time from symptom onset**				
		**0–4 days (n = 75)**	**5–10 days (n = 34)**	**11–21 days (n = 69)**	**22–89 days (n = 31)**	**> 89 days (n = 56)**
**IgM**	Median	0.06	11.16	26.51	23.36	4.59
	IQR	0.02–0.23	2.65–17.92	22.55–28.24	4.55–26.21	2.80–8.75
**IgG**	Median	0.05	0.22	8.94	12.09	13.40
	IQR	0.03–0.09	0.09–2.63	7.67–9.98	10.01–12.96	13.02–13.71

Table S3. Concordance of the VIDAS® CHIKV assays with the respective competitor ELISA according to the time from symptom onset (n=355 samples for anti-IgM assays, n=398 samples for anti-IgG assays [Figure 1]; see Table 6 for the concordance in the whole study population)

VIDAS® CHIKV assay	Time from symptom onset		Positive percent agreement (PPA)	Negative percent agreement (NPA)	Overall percent agreement (OPA)
IgM	0–6 days	*n/N* [1] (%)	76/80 (95.0%)	129/129 (100.0%)	205/209 (98.1%)
		[95% CI]	[87.7–98.6]	[97.2–100.0]	[95.2–99.5]
	7–10 days	*n/N* [1] (%)	17/17 (100.0%)	22/22 (100.0%)	39/39 (100.0%)
		[95% CI]	[80.5–100.0]	[84.6–100.0]	[91.0–100.0]
	11–21 days	*n/N* [1] (%)	51/51 (100.0%)	8/8 (100.0%)	59/59 (100.0%)
		[95% CI]	[93.0–100.0]	[63.1–100.0]	[93.9–100.0]
	22 days–3 months	*n/N* [1] (%)	10/10 (100.0%)	32/32 (100.0%)	42/42 (100.0%)
		[95% CI]	[69.2–100.0%]	[89.1–100.0]	[91.6–100.0]
	> 3 months	*n/N* [1] (%)	3/3 (100.0%)	3/3 (100.0%)	6/6 (100.0%)
		[95% CI]	[29.2–100.0%]	[29.2–100.0%]	[54.1–100.0%]
IgG	0–6 days	*n/N* [1] (%)	28/29 (96.6%)	129/129 (100.0%)	157/158 (99.4%)
		[95% CI]	[82.2–99.9]	[97.2–100.0]	[96.5–100.0]
	7–10 days	*n/N* [1] (%)	10/10 (100.0%)	22/22 (100.0%)	32/32 (100.0%)
		[95% CI]	[69.2–100.0%]	[84.6–100.0]	[89.1–100.0]
	11–21 days	*n/N* [1] (%)	69/69 (100.0%)	8/8 (100.0%)	77/77 (100.0%)
		[95% CI]	[94.8–100.0]	[63.1–100.0]	[95.3–100.0]
	22 days–3 months	*n/N* [1] (%)	90/90 (100.0%)	31/32 (96.9%)	121/122 (99.2%)
		[95% CI]	[96.0–100.0]	[83.8–99.9]	[95.5–100.0]
	> 3 months	*n/N* [1] (%)	6/6 (100.0%)	3/3 (100.0%)	9/9 (100.0%)
		[95% CI]	[54.1–100.0%]	[29.2–100.0%]	[66.4–100.0%]

[1] n/N is the ratio of the number of samples for which VIDAS® assays are in agreement (positive, negative and overall) with the competitor ELISA (comparative method) to the number of samples tested either positive or negative (and overall) with the competitor ELISA. Abbreviations: CI, confidence interval.

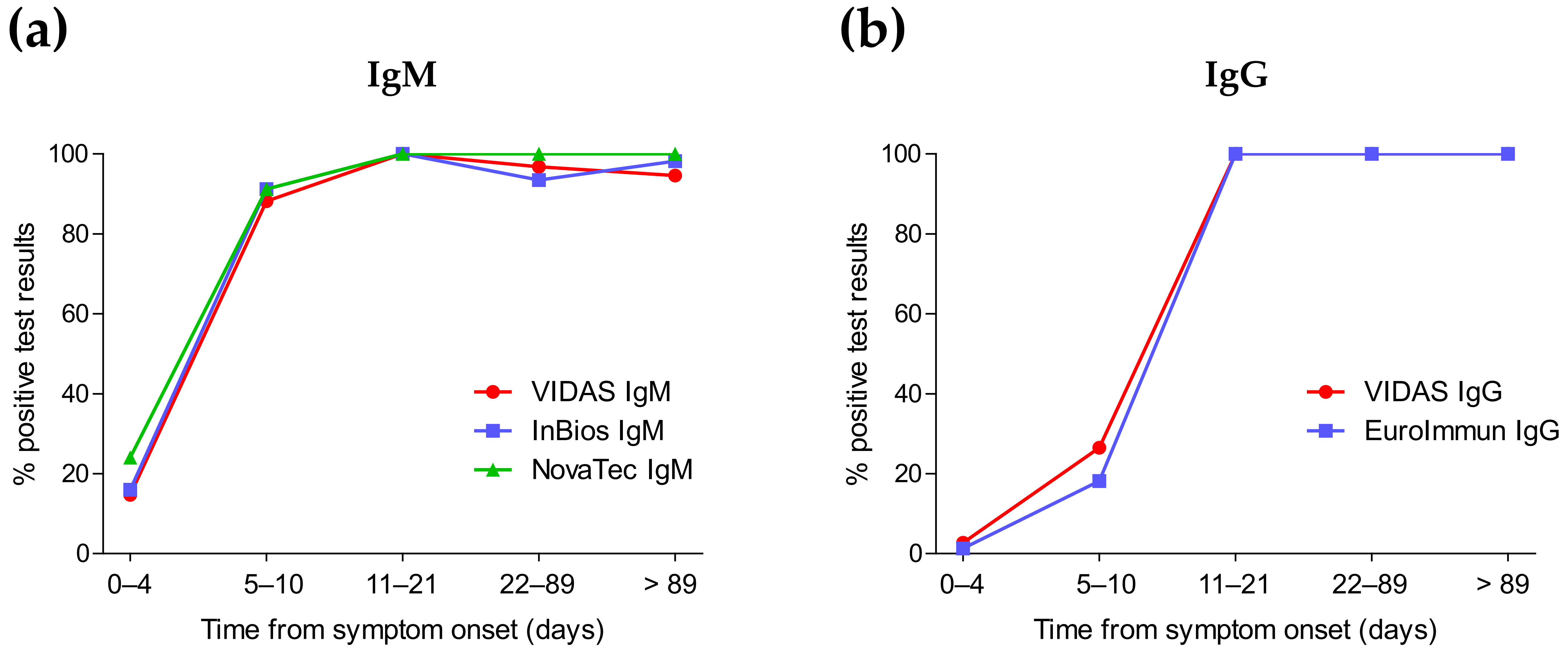

Figure S1. Sensitivity of the VIDAS and competitor ELISA CHIK IgM (**a**) and CHIK IgG (**b**) assays, calculated as the percentage of positive test results among follow-up samples tested positive for RT-PCR within 7 days of symptom onset. Respective percentages and 95% confidence intervals are shown in Table 5.
